# Prognostic value of preoperative serological biomarkers in patients undergoing cytoreductive surgery for ovarian cancer peritoneal metastases

**DOI:** 10.1515/pp-2022-0199

**Published:** 2023-05-16

**Authors:** Charif Khaled, Antoine El Asmar, Omar Raisi, Michel Moreau, Laura Polastro, Isabelle Veys, Florin C. Pop, Vincent Donckier, Gabriel Liberale

**Affiliations:** Department of Surgical Oncology, Jules Bordet Institute - The Brussels University Hospital, Université Libre de Bruxelles (ULB), Brussels, Belgium; Department of Statistics, Jules Bordet Institute - The Brussels University Hospital, Université Libre de Bruxelles (ULB), Brussels, Belgium; Department of Medical Oncology, Jules Bordet Institute - The Brussels University Hospital, Université Libre de Bruxelles (ULB), Brussels, Belgium

**Keywords:** biomarkers ratio, lymphocytes, monocytes, ovarian cancer, peritoneal carcinomatosis, platelets

## Abstract

**Objectives:**

Peritoneal metastases of ovarian cancer (PMOC) are common at initial presentation. Cytoreductive surgery (CRS) of curative intent has been proven to be efficient in increasing the overall survival (OS) and the disease-free survival (DFS) of these patients. Nevertheless, CRS is associated with high postoperative morbidity, which makes patient selection a major concern. Appropriate prognostic factors that can predict patient outcomes after surgery are still lacking. Preoperative biomarkers and their ratios have been shown to be predictive of patient prognosis for various solid tumors. We aimed to study their correlation with the prognosis of patients undergoing CRS for PMOC.

**Methods:**

This retrospective study included patients with PMOC operated by CRS. Preoperative biomarkers and other clinicopathological characteristics were studied to determine their prognostic value in terms OS and DFS.

**Results:**

216 patients were included. Patients with preoperative hemoglobin (Hb) <11.7 g/dL had a poorer prognosis in terms of OS (p=0.0062) and DFS (p=0.0077). Additionally, increased neutrophil-to-lymphocyte ratio (NLR), monocyte-to-lymphocyte ratio (MLR) >0.32, and platelet-to-lymphocyte ratio (PLR) >214.5 were associated with worse OS (p=0.022, p=0.0028, and p=0.0018, respectively) and worse DFS (p=0.028, p=0.003, and p=0.019, respectively). Multivariate analysis showed that the variables mentioned above were independent predictive factors for OS and DFS.

**Conclusions:**

Preoperative Hb level, NLR, MLR, and PLR are prognostic factors for OS and DFS in PMOC patients operated by curative CRS.

## Introduction

Ovarian cancer (OC) is the 8th most common cancer in women. It accounts for 1 % of all new cancer cases. Nevertheless, OC accounts for half of the mortalities related to gynecological malignancies, and the 5-year survival rate is less than 50 %, as over 75 % of new cases are diagnosed at an advanced stage [1]. Upfront or interval cytoreductive surgery (CRS) accompanied by systemic chemotherapy, ± perioperative chemotherapy, is the standard treatment [2]. The extent of residual disease after CRS is one of the most important prognostic factors and indicators of disease-free survival (DFS) and overall survival (OS) [3]. Moreover, elevated cancer antigen 125 (CA125) levels, tumor grade, and malignant ascites have also been proven to predict the prognosis of the disease [4].

In the last two decades, scientists have been investigating biomarkers to search for new cancer prognostic factors. Forrest introduced the Glasgow prognostic score (GPS) in 2004, followed by modified GPS [5]. This score is based on C-reactive protein (CRP) and albumin levels to predict tumor aggression and patient prognosis. It was first tested in patients with non-small cell lung cancer patients [5]. It was later attributed to several other solid organ tumors, such as renal cell carcinoma, gastric adenocarcinoma, and ovarian cancer [5].

Additional research has shown that inflammatory markers can help the development, progression, and spread of tumors by suppressing the proper functioning of the innate immune system [4]. In other words, the inflammatory status plays a major role in the oncological outcomes of patients with various types of cancers [6], [7], [8]. Therefore, researchers have focused on inflammatory markers and, more specifically, their ratios. These included the neutrophil-to-lymphocyte ratio (NLR), monocyte-to-lymphocyte ratio (MLR), and platelet-to-lymphocyte ratio (PLR) [4, 6, 7].

Nevertheless, very few studies have analyzed the relationship between preoperative biomarkers in patients with peritoneal metastases of ovarian cancer (PMOC) undergoing CRS with curative intent, and postoperative prognosis.

This study aims to explore the correlation between prognosis in terms of OS and DFS and several of these biomarkers and their ratios.

## Patients and methods

### Study design and data collection

This retrospective study included patients with PMOC who underwent CRS ± neoadjuvent chemotherapy (NAC) ± hyperthermic intraperitoneal chemotherapy (HIPEC) between 2011 and 2020 at the Jules Bordet Institute. Data was extracted from our database on ovarian cancer and from our institutional medical file system (Oribase). The study was approved by the Institutional Ethics Committee (CE3414).

### Inclusion criteria and variables

The data of patients who underwent CRS ± NAC ± HIPEC for PMOC were analyzed. We only included patients who had no other known malignancies and those who underwent an elective operation. Of those, we only included patients who underwent CRS with curative intent, also defined as completeness of cytoreduction (CCR) 0 and 1.

So, we excluded six patients who underwent palliative R2b resection. We also excluded 10 patients with biological markers measured over 2 weeks before surgery and/or within 2 weeks or less from the last chemotherapy session.

Demographic, clinical, and pathological variables included age, body mass index (BMI), breast cancer gene 1 (*BRCA1*) mutation (when available), NAC and adjuvant chemotherapy, HIPEC, peritoneal disease extent expressed by the peritoneal carcinomatosis index (PCI), pathological (postoperative and post NAC) International Federation of Gynecology and Obstetrics (pFIGO) stage, tumor type, and tumor differentiation. Preoperative biological markers were chosen (post NAC when applicable). These included preoperative CA-125, hemoglobin (Hb), neutrophils, lymphocytes, monocytes, platelets, CRP, and albumin.

### Statistical analysis

Statistical analyses were performed using SPSS version 24. The data were summarized using standard descriptive statistics. Univariate analysis was performed using the Kaplan Meier (KM) test and COX linear regression model to determine the association between our variables and biomarker ratios, OS from the day of diagnosis, and DFS from the day of surgery. Ratio thresholds were defined using the 25th, 50th, and 75th percentiles (p25, p50, and p75). Multivariate Cox regression analysis was then performed, and variables with a p-value <0.1 (on univariate analysis) were studied to rule out their dependency.

## Results

### Demographics and clinicopathological variables

From 2011 to 2020, 232 females underwent elective CRS ± HIPEC for PMOC. Sixteen patients were excluded: six due to palliative R2b resections and ten due to incomplete files. Table 1 summarizes the results regarding the medians and percentages of the demographic and clinicopathological variables. The median age of the patients was 60 years with a mean of 75 years. Most patients (68 %) received NAC, and its indications were based on the primary exploration upon diagnosis; very high PCI (>20) and/or the patient requiring several gastrointestinal resections. Only 11 % had HIPEC. These results are because our patients often presented at an advanced stage; 50 % had a PCI >8. Table 1 shows that only 21.8 % of patients had pFIGO stages I and II, and these were patients who were initially FIGO stages III or IV but responded very well to NAC. The remaining patients (78.2 %) were pFIGO stages III and IV and either partially responded to NAC or did not receive any neoadjuvent treatment. This can be explained by the fact that 69 % of the tumors were poorly differentiated.

**Table 1:** Demographic and clinicopathological variables for patients with PMOC.

Characteristics	n (%)
Median age, years	60
Median BMI, kg/m^2^	24
Median PCI	8
pFIGO stage	
I–II	47 (21.8 %)
III	142 (65.7 %)
IVa	15 (6.9 %)
IVb	12 (5.6 %)
Ovarian tumor histology	
Serous	184 (85.2 %)
Other or mixed	32 (14.8 %)
Degree of differentiation	
Well	35 (16.2 %)
Moderate	32 (14.8 %)
Poor	149 (69 %)
*BRCA1* gene mutation	
Yes	28 (13 %)
No	64 (29.6 %)
Missing	124 (57.4 %)
Median preoperative CA125, U/mL	29
NAC	
Yes	147 (68.1 %)
No	69 (31.9 %)
HIPEC	
Yes	24 (11.1 %)
No	192 (88.9 %)

The median OS of the patients was 68.4 months (5.7 years) from the time of diagnosis, with a five-year overall survival rate of 60 % and median DFS was 26.4 months (2.2 years) from the time of surgery.

### Clinical and biological variables association with OS and DFS

Preoperative albumin and inflammatory biomarkers alone (neutrophils, lymphocytes, monocytes, platelets, and CRP) were not significantly associated with OS or DFS (Table 2).

**Table 2:** Univariate analysis showing the prognostic value of preoperative clinical and serological biomarkers, in terms of OS and DFS, in patients with PMOC.

Variables	Median	OS		DFS	
		Median OS, months	p-Value	Median DFS, months	p-Value
PCI	<7	102	<0.0001	43.5	<0.0001
	>7	55.5		19.2	
pFIGO stage	I–II	115.6	0.002	102	<0.0001
	III–IV	63.7		24.5	
CA125	<35	104	0.034	49	0.003
	>35	64.8		24.9	
Hb	<11.7	60.6	0.006	23.2	0.007
	>11.7	102		31.4	
Neutrophils, ×10^3^/μL	<4.06	68.4	0.72	29.8	0.93
	>4.06	68.5		26.5	
Lymphocytes, ×10^3^/μL	<2.03	64.8	0.18	25.5	0.73
	>2.03	73.92		29.7	
Monocytes, ×10^3^/μL	<0.31	85.4	0.27	30.1	0.25
	>0.31	62.4		26.5	
Platelets, ×10^3^/μL	<264	69.6	0.67	30	0.97
	>264	64.8		24.2	
CRP, mg/L	<2.9	62.5	0.89	30	0.54
	>2.9	67.2		21.6	
Albumin, g/L	<42	64.8	0.57	23.16	0.84
	>42	73.9		26.4	

In contrast, peritoneal burden disease expressed by the PCI was significantly correlated with OS and DFS (Table 2). Patients with a PCI<7 had almost double the OS and DFS medians, compared to those with a PCI ≥7: 102 and 43.5 months, vs. 55.5 and 19.2 months respectively (p<0.0001).

Patients with an earlier pFIGO stage had better chances of survival than patients with a more advanced stage (Table 2). Patients with pFIGO stages I or II had higher median OS and DFS than those with stages III and IV (median OS 115 vs. 63 months, respectively, p=0.0021; median DFS 102 vs. 24 months, respectively, p<0.0001).

Moreover, patients with a preoperative CA125<35 U/mL had a better prognosis than those with a preoperative CA125≥35 U/mL (Table 2): median OS=104 vs. 64 months, respectively; p=0.034, and median DFS=49 vs. 25 months, respectively; p=0.0037).

Finally, patients with preoperative Hb <11.7 g/dL had a poorer prognosis in terms of OS (61 vs. 102 months, p=0.0062) and DFS (23 vs. 31 months, p=0.0077).

### Biological biomarkers ratios and their association with OS and DFS

The CRP/albumin ratio did not correlate with OS or DFS (Table 3).

**Table 3:** Univariate analysis of preoperative inflammatory biomarkers’ ratios, in terms of OS and DFS, using the Cox model.

Ratios	OS		DFS	
	HR (95 % CI)	p-Value	HR (CI at 95 %)	p-Value
NLR (cont)	1.09 (1.01–1.17)	0.022	1.09 (1.01–1.15)	0.028
MLR (p50=0.32)	0.47 (0.29–0.77)	0.0028	0.54 (0.36–0.81)	0.003
PLR (p75=214.5)	0.7 (0.46–1.05)	0.0018	0.63 (0.43–0.93)	0.019
CRP/Alb (cont)	0.98 (0.92–1.05)	0.63	1 (0.95–1.06)	0.86

Conversely, preoperative NLR, MLR, and PLR were significantly associated with OS and DFS in the univariate analysis using the Cox model (Table 3). NLR was negatively associated with OS and DFS. As the NLR increased, OS and DFS decreased (p=0.022 and p=0.028, respectively). Nevertheless, no cutoff value was found using the KM model.

Regarding MLR, a cut-off at the 50th percentile (p50=0.32) revealed a clinically significant correlation between lower MLR and better prognosis in terms of OS and DFS (p=0.0053 and p=0.044, respectively) (Table 3). This was further confirmed using the Cox model analysis with HR=0.47 and 95 % CI 0.29–0.77 (p=0.0028) for OS, and HR= 0.54 and 95 % CI 0.36–0.81 for DFS (p=0.003). And we found that patients with MLR≤0.32 had a median OS of 102 months, while those with MLR>0.32 had a median OS of 60 months.

As for PLR, a cutoff value at the 75th percentile, p75=214.5, was significant for OS and DFS (Table 3). The higher the PLR, the worse the OS and DFS (p=0.0018 and p=0.019, respectively).

### At multivariate analysis using the cox model

Several variables were analyzed using a multivariate Cox model. Those that proved to be independent predictors of both OS and DFS in patients with PMOC are PCI, pFIGO stage, Hb, NLR, MLR, and PLR (Table 4).

**Table 4:** Multivariate analysis of clinical and serological biomarkers, using the Cox model, in patients with PMOC.

Ratio	OS		DFS	
	HR (95 % CI)	p-Value	HR (95 % CI)	p-Value
PCI <7	0.41 (0.27–0.61)	<0.0001	0.37 (0.26–0.53)	<0.0001
pFIGO Stage I–II	0.33 (0.15–0.71)	0.005	0.32 (0.16–0.63)	0.001
Hb <11.7	1.62 (1.11–2.38)	0.014	1.51 (1.07–2.14)	0.019
NLR (cont)	1.09 (1–1.17)	0.021	1.07 (1–1.14)	0.04
MLR ≤0.32	0.47 (0.29–0.77)	0.003	0.53 (0.35–0.8)	0.002
PLR ≤214.5	1.02 (1–1.03)	0.003	0.65 (0.44–0.96)	0.032

## Discussion

Our study focused on the prognostic value of preoperative biomarkers (Hb, albumin, CRP, neutrophils, lymphocytes, monocytes, and platelets), and more specifically, inflammatory biomarkers and their ratios (NLR, MLR, and PLR). In this study, we report that NLR, MLR, and PLR are independent prognostic factors for OS and DFS in patients with PMOC. Moreover, as expected, factors such as preoperative CA-125 level, PCI, pFIGO stage, and preoperative Hb level were prognostic factors for OS and DFS. However, the preoperative values of neutrophils, lymphocytes, monocytes, albumin, and CRP were not associated with OS or DFS.

PCI, pFIGO stage, and preoperative CA-125 often reflect the tumor and disease extent and are therefore associated with patient prognosis. PCI is one of the most important factors in predicting OS in patients with PM [9]. Llueca and Escrig showed that PCI >10 is strongly associated with a worse prognosis [10]. This is similar to our results, which show a better prognosis for patients with PCI <7. The FIGO staging system also provides accurate information regarding prognosis, and guides the management of ovarian cancer [11]. As stages III and IV reflect advanced disease, it is natural for patients with stages I and II on final pathology to have a better prognosis, especially since these patients are ones that responded well to NAC. Nevertheless, a limited number of studies have explored the prognostic value of pathological response to NAC, and most of these studies only correlated complete pathological response with a better prognostic outcome [12]. As for CA-125, despite having high false-positive rates [13], it has long been known for its predictive value in the prognosis of ovarian cancer [13], [14], [15]. Our results correlate with those reported in several studies and show that patients with preoperative CA-125 levels <35 have a better prognosis and a lower risk of disease recurrence.

In recent years, several studies have shown the significance of preoperative serological and inflammatory biomarkers and their ratios in predicting prognosis in different solid tumors (stomach, colon, ovarian, lung, breast and so on) [16], [17], [18]. Nevertheless, very few studies have evaluated the value of these ratios in patients undergoing CRS for PMOC, which leads us to the focus of the present study, which is the evaluation of the association between preoperative serological biomarkers and their ratios with prognostic outcomes in patients undergoing CRS for PMOC.

Regarding the preoperative Hb rate, cancer patients’ quality of life is substantially impacted by anemia [19], and many patients are anemic following chemotherapy, surgery, or even bleeding tumors, metastasis, or inflammatory status [20]. Abu-Zaid et al. showed that a low preoperative Hb level (<12 g/dL) correlates well with advanced FIGO stage III/IV, positive peritoneal cytology, and lymph node metastasis for endometrial cancer [20]. Similarly, to our results, they reported that the 5-year OS and DFS rates were reduced in patients with preoperative anemia. In addition, Chen et al. reported similar results [21] showing that preoperative hematocrit <35 % (equivalent to a Hb of 11.7 g/dL) in patients with ovarian cancer undergoing CRS predicted poorer OS and DFS, especially in those with FIGO stage III and IV.

NLR has been proven to be a prognostic factor for OS and DFS in patients undergoing curative CRS [7]. Our study showed that the higher the ratio, the worse the OS and DFS outcomes. In their systematic review and meta-analysis, Templeton et al. reported that a high NLR pre-treatment and/or pre-surgery correlates poorly with outcomes in solid tumors, including ovarian cancer [16]. Williams et al. observed similar results and explained that an elevated NLR indicates a more aggressive disease [17]. Finally, Yang et al. concluded that the preoperative NLR predicts the prognosis of patients with ovarian cancer [22]. This phenomenon can be explained by the fact that neutrophils secrete vascular endothelial growth factor (VEGF), leading to tumor angiogenesis. Therefore, a higher neutrophil count and a lower lymphocyte count reflects higher tumoral activity and angiogenesis.

Regarding MLR, the higher the ratio, the poorer the prognosis (cutoff 0.32). Lower cut-offs have been reported in the literature, respectively, “0.23” [24] and “0.26” [24], but with the same significant value. The significance of the MLR ratio is explained by leukopenia, as discussed previously, and by monocytosis. Experimental studies have shown that monocytes differentiate into tumor-associated macrophages after entering the tumor tissue. These tumor-associated macrophages enhance angiogenesis, matrix breakdown, and tumor cell motility, all of which facilitate the metastatic process [23].

It has been reported that a high PLR correlates with poor survival for various cancers, including ovarian cancer [16, 25, 26]. In our study, we reported a cut-off PLR of 214.5, which borders those reported in the literature (210 [4] and 226 [25], respectively). Asher et al. attributed this to systemic inflammation, which leads to suppression of the immune system and reduction of lymphocyte function [26]. Moreover, inflammation causes thrombocytosis, which leads to the activation of urokinase plasminogen activator (uPA) and VEGF, eventually increasing the malignant progression of ovarian cells [16]. Therefore, leukopenia and thrombocytosis are consequences of ovarian cancer and lead to deleterious effects and aggressive disease. These results demonstrate the impact of biological markers compounding the immune system on cancer progression.

Preoperative serological biomarker ratios, including NLR, MLR, and PLR, are independent variables that predict OS and DFS in patients treated for PMOC by curative CRS.

However, this study was limited by its retrospective design. Moreover, we cannot exclude certain factors not related to OC per se and its treatment, which might have influenced our results, such as existing medical comorbidities and certain medications. Further investigations are required in prospective studies with larger exclusion criteria.

## Conclusions

Our study concluded that a simple complete blood count can be a prognostic indicator for patients with PMOC undergoing curative-intent CRS. Preoperative Hb level, NLR, MLR, and PLR were significant independent prognostic variables for OS and DFS. Such inexpensive and easily accessible biomarkers can be used, in addition to other parameters such as PCI, pFIGO stage, and preoperative CA125 level, to determine the prognosis of patients with PMOC. This can help oncologists and surgical oncologists to better select patients eligible for CRS and devise individual patient-tailored courses of treatment and postoperative surveillance plan.
